# Topo-Pachimetric Accelerated Epi-On Cross-Linking Compared to the Dresden Protocol Using Riboflavin with Vitamin E TPGS: Results of a 2-Year Randomized Study

**DOI:** 10.3390/jcm10173799

**Published:** 2021-08-25

**Authors:** Ciro Caruso, Robert Leonard Epstein, Pasquale Troiano, Francesco Napolitano, Fabio Scarinci, Ciro Costagliola

**Affiliations:** 1 Corneal Transplant Center, Pellegrini Hospital, Via Portamedina alla Pignasecca, 41, 80127 Napoli, Italy; 2 The Center for Corrective Eye Surgery, W. Elm Street, 5400, Mc Henry, IL 60050, USA; rlepstein@aol.com; 3 Department of Ophthalmology Sacred Family, Fatabenefratelli Hospital, Via Fatebenefratelli, 20, 22036 Erba, Italy; ptroiano@fatebenefratelli.eu; 4 Department of Ophthalmology, Pellegrini Hospital, Via Portamedina alla Pignasecca, 41, 80127 Napoli, Italy; eyenapoli@libero.it; 5 Department of Ophthalmology, IRCCS Fondazione, Bietti, Via Livenza, 3, 00198 Roma, Italy; fabioscarinci@gmail.com; 6 Eye Clinic, Department of Medicine and Health Sciences “V. Tiberio”, University of Molise, Via Francesco De Sanctis, 1, 86100 Campobasso, Italy; ciro.costagliola@unimol.it

**Keywords:** riboflavin, vitamin E TPGS, aCFXL, sCXL

## Abstract

In the present study (clinical trial registration number: NCT05019768), we compared the clinical outcome of corneal cross-linking with either the standard Dresden (sCXL) or the accelerated custom-fast (aCFXL) ultraviolet A irradiation protocol using riboflavin–D-α-tocopheryl poly(ethylene glycol)-1000 succinate for progressive keratoconus. Fifty-four eyes of forty-one patients were randomized to either of the two CXL protocols and checked before treatment and at the 2-year follow-up. The sCXL group was subjected to CXL with 30 min of pre-soaking and 3 mW/cm^2^ UVA irradiation for 30 min. The aCFXL group was subjected to CXL with 10 min of pre-soaking and UVA irradiation of 1.8 ± 0.9 mW/cm^2^ for 10 min ± 1.5 min. In both groups, a solution of riboflavin–vitamin E TPGS was used. Uncorrected distance visual acuity, corrected distance visual acuity, pachymetry, Scheimpflug tomography, and corneal hysteresis were performed at baseline and after 24 months. Both groups showed a statistically significant improvement in corrected distance visual acuity, and keratometric and corneal hysteresis compared to baseline conditions; no statistically significant differences in outcomes between the two groups were observed. Improvement in refractive, topographic, and biomechanical parameters were observed after sCXL and aCFXL, making the riboflavin–VE-TPGS solution an effective option as a permeation enhancer in CXL procedures. Deeper stromal penetration of riboflavin could be complemented by photo-protection against UVA and free radicals formed during photoinduced processes.

## 1. Introduction

Cross-linking (CXL) is a well-known treatment for keratoconus and ectatic cornea disorders, with various authors confirming its effectiveness in slowing or stopping the progression of the disease [1,2,3].

The induction of cross-links between corneal stromal fibrils by photosensitizing riboflavin and UV irradiation provides an increase in corneal stiffness and strength, thus stabilizing the ectatic process [4].

The results obtained with this treatment have shown a reduced need for keratoplasty in patients with keratoconus [5,6].

In recent years, the standard CXL (sCXL) Dresden protocol has been challenged by new therapeutic approaches, such as using higher UVA irradiation values (Table 1) [7].

The rationale for the high-intensity approach is based on the Bunsen–Roscoe reciprocity law, which states that the time and intensity of irradiation can be varied without changing the total radiation energy of 5.4 J/cm^2^ of the standard protocol of Dresden [8,9].

The intensity of 3 mW/cm^2^ in the Dresden protocol was heuristically established; on the contrary, the accelerated custom fast CXL (aCFXL) protocol has been developed on a published mathematical model that takes into consideration objective variables such as the equation governing the UVA-induced riboflavin consumption rate and the corneal thickness at its thinnest point. By knowing both values, the mathematical model allows one to objectively calculate both the exact UVA irradiation value (intensity), expressed in mW/cm^2^, and the irradiation time [10,11,12]. Furthermore, this procedure never exceeds the endothelial safety threshold of 0.35 mW/cm^2^ [13,14]

Using this approach, it was possible to develop a customized, accelerated, pachymetry-dependent CXL protocol that has been named accelerated custom fast corneal cross-linking (aCFXL).

Reducing the UVA exposure time, and therefore the total time of the CXL procedure, can be beneficial for the patient.

So far, different CXL protocols have been proven effective in stopping the progression of keratoconus, with outcomes similar to standard CXL [1,15].

However, it is difficult to compare these studies, because different UVA irradiation profiles were applied [16].

In addition, there is no consensus among physicians about the clinical effectiveness of aCXL protocols in comparison with the sCXL protocol. Indeed, while some studies have found similar results [17,18,19,20], other studies have reported less topographical improvement after aCXL than sCXL [21,22,23].

So far, researchers have focused their attention on improving the clinical outcomes of the aCXL protocol only by modulating the intensity and time of UVA irradiation; conversely, little has been done in testing new and more effective solutions to improve riboflavin solution penetration. Indeed, in most of the studies performed so far, dextran was the only enhancer used. In a comparative study, in which dextran–riboflavin was used, a trend of increased corneal curvature was found in corneas that received aCXL with UVA irradiation at 9 mW/cm^2^ for 10 min compared to standard sCXL. It was hypothesized that the lower clinical outcome after aCXL could be explained by a limited diffusion rate of dextran–riboflavin into the cornea when using a shorter treatment time.

In CXL based on accelerated but not customized UVA profiles, both the UVA irradiation and, therefore, the total soaking time are reduced. This can lead to a lower corneal concentration of riboflavin, which can result in less effective treatments.

In our previous comparative study, the time-dependent corneal accumulation of riboflavin–vitamin E TPGS or a control riboflavin solution was evaluated in both conditions: without epithelial debridement (epi-on) or after debridement (epi-off) [24]. No statistical differences were found between the solution containing the permeation enhancer (vitamin E TPGS (VE-TPGS)) and the control solution, thus demonstrating the efficacy of VE-TPGS–riboflavin solution in overcoming the resistance of the epithelium to corneal permeability.

To improve diffusion, in the present study, a VE-TPGS–riboflavin solution was used instead of the dextran–riboflavin solution.

D-α-tocopheryl poly(ethylene glycol)-1000 succinate (VE-TPGS) is a well-known non-ionic surfactant widely used as a solubilizer, emulsifier, and vehicle for lipid-based drug release formulations. VE-TPGS as a specific riboflavin transporter has been described in the rabbit corneal epithelium [25] and in human-derived retinoblastoma cells [26].

Furthermore, non-ionic surfactants have been widely described as enhancers of ocular permeation, with particular efficacy for the more hydrophilic molecules [27].

VE-TPGS has been proven effective in extinguishing potentially harmful oxidation-inducing substances, such as reactive oxygen species [28].

In recent years, we saw the introduction of VE-TPGS-enriched riboflavin solutions that showed enhanced stromal penetration during CXL’s soaking phase. Riboflavin solution has also shown been to exert corneal endothelial layer protection during the irradiation step of CXL procedures. This feature has been exploited by physicians during the standard CXL procedure; indeed, continued riboflavin corneal wetting during UVA irradiation is mandatory in order to prevent UVA-induced corneal endothelium damage [14,24]. The epithelial layer is, in fact, the first corneal structure irradiated by UVA rays during CXL and consequently absorbs a large amount of radiation. Irradiation-induced damage to epithelial structures is well documented. Starting from this evidence, the aim of this comparative clinical study was to evaluate the safety and efficacy of the use of a riboflavin–VE-TPGS solution as an enhancer of the ocular permeation of the solution and as a photo-protective agent against UVA radiation in both procedures, sCXL and aCFXL UVA irradiation.

The study was a 2-year follow-up, evaluating clinical, visual, refractive, topographic, and biomechanical parameters.

To the best of our knowledge, there is no previous comparative CXL study between standard and different UVA irradiation protocols using riboflavin with VE-TPGS in both protocols.

## 2. Materials and Methods

### 2.1. Study Design and Patients

Based on the literature [29], the sample size was calculated to detect a difference of 0.95 D between the average Kmax changes in aCFXL and sCXL groups at 12 months, at a significance level of 5% and a power of 80%, assuming a standard deviation of 1.20 D. The sample size of the study was 54 observation (t-test for independent groups, two tails, using G*Power software version 3.1.9.7).

We randomized 54 eyes of 41 patients with keratoconus to either sCXL or aCFXL protocols and checked them before treatment and at the 2-year follow-up in this prospective, randomized controlled study. The randomization process was performed by using a cell phone app that was able to generate computerized random numbers. The allocation to either group was performed 1 week before the treatment when the patient was identified as eligible for the study.

This clinical study was conducted according to the ethical standards of the Declaration of Helsinki (revised in 2000). Patients were informed about the nature and purpose of the trial, and they provided informed consent. The approval of the institutional review/ethics committee (IRB) of the Corneal Transplant Center, Pellegrini Hospital, Napoli, Italy, was obtained (authorization 1269/2017). It was not appropriate or possible to involve patients or the public in the design, conduct, reporting, or dissemination plans of our research.

The patients were randomized (1:1) to receive either the standard or the customized protocol. In particular, 29 eyes were allocated to the sCXL group and 25 to the aCFXL group.

The mean age (±SD) of the patients in the sCXL and aCFXL groups was 28 ± 7.5 years and 26.3 ± 8.3 years, respectively. The mean follow-up period was 24.10 ± 3.30 months in the sCXL group and 25.20 ± 2.60 months in the aCFXL group.

The sCXL group underwent conventional UVA of 3 mW/cm^2^ for 30 min, while the aCFXL group underwent the accelerated customized procedure (1.8 ± 0.9 mW/cm^2^ for 10 min ± 1.5 min). The differences in CXL UVA irradiation treatment between the Dresden sCXL group and the aCFXL group are shown in Table 1. The Amsler–Krumeich classification system was used to define inclusion criteria. Furthermore, the treated eyes’ distribution among the different keratoconus stages was as follows: sCXL stage 1 = 4, stage 2 = 16, and stage 3 = 9; aCFXL stage 1 = 3, stage 2 = 14, and stage 3 = 8.

The patients’ eligibility was based on documented progressive keratoconus evaluated on corneal topography with Scheimpflug imaging (Pentacam HR; Oculus, Wetzlar Inc., Wetzlar, Germany) (increase of 1.0 D or more in the steepest keratometry), minimum corneal thickness reduction (thinnest point) of 5% or more on corneal pachymetry values, and changes in uncorrected distance visual acuity (UDVA) and corrected distance visual acuity (BCVA) (cylinder increase above 1.00 D or spherical equivalent greater than 0.50 D). To improve the reliability of topography measurements, a minimum of 3 acquisitions were obtained for each eye at each time interval. Topography evaluation was performed by only one trained operator to reduce inter-observer variation. If the value varied by more than 10% between the scans, then another scan was obtained. The best scan was then selected for analysis.

Visual acuity was measured with a logarithm of the minimum angle of resolution (logMAR) using the Early Treatment Diabetic Retinopathy Study chart at 4 m, and corneal hysteresis (CH) was measured using an Ocular Response Analyzer (ORA, Reichert, Depew, NY, USA). These parameters were evaluated for at least 6 months and up to 1 week before treatment. Patient demographic data with the mean clinical parameters for comparison of baseline characteristics between the Dresden sCXL group and the aCFXL group are shown in Table 2.

We excluded eyes with corneal pachymetry of less than 400 µm at the thinnest point, endothelial cell density (using a Tomey EM-3000 (Tomey Corp, Nagoya, Japan)) of less than 2000 cells/cm^2^, corneal scarring, nystagmus or any motility disorder that prevented a fixed gaze during examination, and other significant pathologies. All patients discontinued contact lens use for at least 3 days prior to the screening visit.

The clinical observations were reported at 6 months and 1 week before treatment and at 1, 3, 6, 12, and 24 months after treatment. A complete ophthalmic examination was performed, including the best-corrected visual acuity (BCVA) with glasses based on the logMAR graph, manifest refraction, slit lamp, and dilated fundoscopy for all patients. Central minimum pachymetry was also measured using the Pentacam system.

The endothelial cell density was assessed using a non-contact specular microscope (Tomey EM-3000, Tomey Corp, Nagoya, Japan).

### 2.2. Surgical Technique

Both CXL procedures were performed under topical anesthesia and in the operating room. In the Dresden sCXL group, corneal pachymetry was performed before the procedure. The corneal epithelium was then partially removed from a treatment area of 9.0 mm using a smooth spatula after applying 10% diluted ethyl alcohol. A drop of 0.1% riboflavin–0.5% VE-TPGS solution (Ribocross, Iromed Group s.r.l., Rome, Italy) was instilled every 2 min for 30 min (15 drops).

Subsequently, the corneal thickness measurement was repeated and, if less than 400 µm, two drops of 0.1% hypotonic riboflavin solution (Ribofast, Iromed Group S.r.l., Rome, Italy) were instilled every 10–15 s until the corneal thickness was at least 400 µm. In this phase, we performed ultrasound thickness measurement with a 5 µ resolution pachymeter (Quantel Medical™, Cournon-d’Auvergne, France). The UV lighting device (CF-X LINKER, Iromed Group S.r.l., Rome, Italy) was the same for both protocols, positioned 5 cm away from the patient’s eye. The riboflavin–vitamin E TPGS solution was then instilled every 2 min during the exposure, with a UV power of 3 mW/cm^2^ for 30 min (total energy: 5.4 J/cm^2^).

In the aCFXL group, a drop of 0.1% riboflavin–0.5% VE-TPGS solution (Ribocross, Iromed Group S.r.l., Rome, Italy) was instilled every 15 s for 10 min without removing the corneal epithelium. The eye was then rinsed thoroughly with a balanced salt solution and aligned under the UV lighting system (CF-X LINKER, Iromed Group S.r.l., Rome, Italy). Before starting the treatment, the corneal thinnest point value was entered into the device, which automatically calculated the treatment time and UV power for each patient. The modulated irradiation thus obtained was carried out at an average intensity of 1.8 ± 0.9 mW/cm^2^ for 10 ± 1.5 min (total energy: 1.08 ± 0.6 J/cm^2^). The main differences between the two protocols are summarized in Table 1. Briefly, in the sCXL protocol, continuous riboflavin–VE-TPGS solution administration on an epithelium-deprived cornea is necessary to better protect the endothelium, as demonstrated by Wallensak et al. [13,14]. Conversely, in the aCFXL protocol, riboflavin–VE-TPGS solution is applied once before UVA radiation without removing the epithelium. In addition, in the aCFXL protocol, the UVA fluency modulation is automatically modulated (using the published algorithm [10,11,12]) by the UV lighting system (CF-X LINKER, Iromed Group S.r.l., Rome, Italy).

In the Dresden sCXL group, a contact lens with a bandage was applied after the procedure; this was not necessary for the accelerated aCFXL group. In both protocols, patients took topical antibiotics (0.5% moxifloxacin hydrochloride) and steroids (0.12% prednisolone acetate); in the standard protocol, the therapy was gradually reduced during the first month after complete epithelial healing, while it only took 3 days for the customized protocol. All patients were examined after the procedure at 1 day, 1 week, and 1, 3, 6, 12, and 24 months.

### 2.3. Statistical Analysis

The normal data distribution was tested using the one-sample Kolomogorov–Smirnov test. Data were analyzed by one-way ANOVA for repeated measures, while Bonferroni correction was used to adjust for multiple comparisons. A probability of less than 5% (*p* < 0.05) was considered statistically significant. SPSS was the software used.

## 3. Results

Baseline characteristics showed no significant differences between the two groups (Table 2); furthermore, eye distribution among the different stages was non-statistically significant.

### 3.1. Refractive Parameters

Compared to baseline conditions, the BCVA improved significantly in both groups at the 12- and 24-month follow-up (one-way ANOVA for repeated measures: f = 16.36; *p* < 0.0001; sCXL group: 12 months, *p* = 0.04; 24 months, *p* = 0.02; aCFXL group: 12 months, *p* = 0.05; 24 months, *p* = 0.012; Table 3).

Similarly, the spherical equivalent improved at the 12- and 24-month follow-up in both groups (one-way ANOVA for repeated measures: f = 110.56; *p* < 0.0001). In particular, in the aCFXL group, the spherical equivalent had a *p*-value of 0.0012 at 12 months and *p* = 0.006 at 24 moths; the sCXL group showed a *p*-value of 0.02 at 24 months.

In contrast, while a significant decrease in cylinder correction was observed at both 12 and 24 months in the aCFXL group (one-way ANOVA for repeated measures: f = 96.22; *p* < 0.0001; *p* = 0.02 and *p* = 0.014, respectively), in the sCXL group, significant cylinder correction occurred only after 24 months (*p* = 0.02).

No significant difference was found for the analyzed parameter between 12 months and 24 months.

Intergroup analysis showed no statistically significant differences.

### 3.2. Topographic Parameters

Both Kmax and Kmean significantly improved in both groups at 12 and 24 months (one-way ANOVA for repeated measures: f = 86.31 and *p* < 0.0001 for Kmax and f = 61.05 and *p* < 0.0001 for Kmean; Table 3). For both groups, no statistical difference was observed in the minimum corneal thickness.

Intragroup comparison at 12 and 24 months showed no statistically significant differences.

Intergroup analysis showed no statistically significant differences.

### 3.3. Corneal Biomechanical Parameters

The CH significantly decreased in both groups at both 12 and 24 months (one-way ANOVA for repeated measures: f = 106.43; *p* < 0.0001; aCFXL: *p* = 0.002 at 12 months and *p* = 0.008 at 24 months; sCXL: *p* = 0.004 at 12 months and *p* = 0.021 at 24 months).

No significant differences were found between 12 and 24 months in both groups.

Intergroup analysis showed no statistically significant differences between groups.

### 3.4. Complications

Two patients in the sCXL group developed late-onset deep stromal scarring. In both cases, the stromal scar formation was located far from the visual axis and did not affect the final best spectacle-corrected visual acuity. There were no long-term complications in the aCFXL group.

## 4. Discussion

The results of this 2-year follow-up study of CXL treatment demonstrated that by using VE-TPGS-enriched riboflavin, it is possible to obtain overlapping clinical results using both surgical techniques: aCFXL and sCXL. Indeed, both groups showed statistically significant improvements in BCVA, Kmax, and CH parameters, and there were no significant differences in the observed changes between the two groups.

Currently, there is no agreement on the safety and effectiveness of aCFXL compared with the standard Dresden protocol. In this study, both groups showed improvement in the BCVA (logMAR) at 12 and 24 months after treatment.

An in vitro CXL study showed a lower biomechanical stiffening effect in high-UV-power accelerated protocols in porcine corneas [30].

The group that received the highest UV intensity (18 mW/cm^2^ for 5 min) had the same stiffness values as the control group after the treatment. Since oxygen is required for covalent bond formation through a photo-oxidative reaction, this study hypothesized that oxygen depletion is the reason behind the subsidiary effect of accelerated CXL [31,32].

Oxygen depletion could be caused by the disparity between its diffusion capacity and its consumption in the corneal stroma with greater irradiation.

Using customized accelerated UVA irradiation profiles with the same riboflavin–vitamin E TPGS solution led to a significant improvement in keratometric outcomes after both aCFXL and sCXL treatments [33,34].

The results described in our study further highlight the ability of CXL (both sCXL and aCFXL) to improve the CH, an effect that remains constant till after 24 months.

The CH is a biomechanical parameter together with the corneal resistance factor (CRF). The CH is an indicator of corneal viscosity, while the CRF represents the cornea’s ability to counteract deformation [35,36].

The ORA system is a non-contact applanation tonometer that assesses the corneal response to indentation (change in shape) induced by an air pulse.

CXL-induced CH variation has been studied by several authors, with conflicting results. While Sedaghat et al. [37], Goldich et al. [35], and Spoerl et al. [38] reported little to no variation in both the CH and the CRF, authors such as Lanchares et al. and Wollensak et al. [39,40] highlighted an increased corneal rigidity following CXL. These conflicting results can be explained by taking into consideration two main factors: one methodological and one pathology linked. Regarding the methodological factor, Sedaghat et al. [37], Goldich et al. [35], and Spoerl et al. [38] used the ORA in vivo system; on the contrary, Lanchares et al. and Wollensak et al. [39,40] used the strip extensometry in vitro system.

The pathology-related factor is the non-homogeneous corneal curvature. This implies that taking the mean of the variable measurements may lead to neglect the subtle changes in the CH and CRF [8]. Regarding this, it is worth noting that Spoerl and coworkers reported conflicting results in the same study by measuring the CH and CRF at different points on the cornea [38].

This suggests that the ORA is not able to measure the cross-linking-induced changes in the CH and CRF. Indeed, the ORA measures the biomechanics of collagen fibers and the viscous ground substance (proteoglycans and glycosaminoglycans), while CXL changes only collagen fibers. Therefore, using a static method may provide a better evaluation of the CXL effects on the cornea [9,10]. Despite the abovementioned limitations, we collected and reported the ORA CH data for two main reasons: First, we aimed to provide a complete picture of the differences we identified; second, the ORA is the only system able to provide such data in vivo. However, the validation of such procedure is out of the scope of this study.

Our findings, together with the already published ones, become even more relevant if we consider that sCXL and non-customized aCXL procedures usually involve removal of the corneal epithelium. Indeed, the epithelium prevents the passage of riboflavin, limiting the concentration of the molecule in the corneal stroma and, therefore, the effectiveness of the treatment. At the same time, low concentrations of stromal riboflavin increase the risk of tissue photodamage after UVA irradiation [14].

Removal of the corneal epithelium causes pain, ocular photophobia, and transient blurring. These symptoms persist until the corneal epithelium has been restored. In addition, the use of lubricating eye drops and antibiotics, analgesic oral therapy, and therapeutic contact lenses is necessary for healing. Occasionally, complications such as infections, keratitis, edema, and corneal scarring may arise, potentially leading to further loss of vision. Corneal thickness is also an essential parameter in CXL treatments. UV damage to deeper structures, especially the endothelial layer, is even more likely in thin corneas. It is known that the minimum safe corneal thickness to protect against endothelial damage is 400 µm [41].

Unfortunately, many patients with progressive keratoconus have corneas thinner than this threshold and, therefore, are excluded from treatment. Since the thickness of the human corneal epithelium is reported to be around 50 µm [42], avoiding the removal of the epithelium can allow for more patients to be treated. Several approaches have been described so far to overcome these problems. Hyposmolar riboflavin solutions have been used to swell the corneal stroma by more than 400 µm to treat thinner corneas, but without consistent evidence. Other procedures have been described as treatment of thin corneas, such as the accelerated cross-linking nomograms, the sub-400 protocol, the M nomogram for standardized treatment of thin corneas, contact-lens-assisted treatments, and the smile-lenticule-assisted epi-off CXL [43,44,45].

The introduction of the aCFXL protocol avoids debridement of the corneal epithelium, while increasing the corneal concentration of riboflavin by using corneal permeation stimulators such as VE-TPGS.

Since data from the present study were collected using only one type of equipment, different type of cameras might provide different results; therefore, the date described in the present manuscript might be not translated on different equipment [46].

## 5. Conclusions

In conclusion, this is the first study comparing 2-year outcomes after standard sCXL and aCFXL protocols customized using riboflavin–VE-TPGS in both irradiation profiles.

Since both study groups achieved similar clinical results, we can conclude that using riboflavin–VE-TPGS solution during both sCXL or aCFXL is a safe and effective approach. Furthermore, since it has been already shown that vitamin E is an effective candidate as a permeation enhancer in CXL procedures, we can also speculate that riboflavin–VE-TPGS solution might also positively impact the CXL procedure. Indeed, due to a deeper stromal penetration of riboflavin, more efficient photo-protection against UVA rays and free radicals formed during photoinduced processes can be achieved. Larger, prospective, randomized controlled trials with longer follow-ups are now necessary to confirm the long-term safety and efficacy of riboflavin–VE-TPGS solution in CXL with different irradiation profiles.

## Figures and Tables

**Table 1:** Different cross-linking protocols.

Differences among sCXL, aCXL, and aCFXL			
	sCXL	aCFXL	aCXL
Epithelium removal	Yes	No	Yes/No
Soaking time	30 min	10 min	Variable
Pre-soak eye drop frequency	Every 2 min	Every 15 s	Every 2 min
UVA fluence	3 mW/cm^2^	1.8 ± 0.9 mW/cm^2^	9 to 45 mW/cm^2^
UVA fluence variation	Unchanging	Pachymetry and time dependent algorithm; fluence declining during treatment	Unchanging
Riboflavin during UVA	Yes; every 2 min	No; epithelial lavage before UVA	Yes; every 2 min
UVA irradiation time	30 min	10 ± 1.5 min	2 to 15 min
UVA irradiation method	Non-pulsed	Pulsed	Pulsed

Legend: sCXL, standard Dresden CXL; aCFXL, accelerated custom fast CXL; aCXL, accelerated CXL.

**Table 2:** Comparison of baseline parameters in the sCXL and aCFXL groups.

	sCXL (*n* = 29)	aCFXL (*n* = 25)	*p*-Value
Age years	28 ± 7.5	26.3 ± 8.3	0.432
Male/female eyes	18:11	15:10	0.194
**Refractive parameters**			
Corrected distance acuity (logMAR) (BCVA)	0.12 ± 0.12	0.10 ± 0.13	0.504
Spherical equivalent (D)	−4.11 ± 3.07	−4.70 ± 3.38	0.172
Refractive cylinder (D)	3.47 ± 1.35	3.25 ± 1.58	0.323
**Topographical parameters**			
Maximum keratometry (D) (6 months preoperative)	47.92 ± 5.22	47.65 ± 5.22	0.902
Maximum keratometry (D) (1 week preoperative)	49.93 ± 5.20	49.64 ± 5.20	0.326
Mean K (D) (6 months preoperative)	48.52 ± 4.17	46.82 ± 4.67	0.336
Mean K(D) (1 week preoperative)	49.48 ± 4.02	47.83 ± 4.66	0.184
Minimum corneal thickness (µm) (6 months preoperative)	435.0 ±54.8	461.0 ±56.3	1.000
Minimum corneal thickness (µm) (1 week preoperative)	449.0 ± 51.9	456.0 ± 56.6	0.582
**Biomechanical parameters**			
Corneal hysteresis (mmHg) (1 week preoperative)	7.91 ± 1.1	8.32 ± 1.7	0.676
Corneal resistance factor (mmHg) (1 week preoperative)	6.42 ± 1.3	6.58 ± 1.5	0.595
Endothelial cell density (cells/mm^2^) (1 week preoperative)	2586 ± 246	2549 ± 263	0.184
Follow-up (months)	24.10 ± 3.30	25.20 ± 2.60	0.432

Legend: BCVA, best-corrected visual acuity; D, diopter; logMAR, logarithm of the minimum angle of resolution.

**Table 3:** Mean changes (postoperative values–preoperative values) of clinical outcomes in sCXL and aCFXL groups (*n* = studied eyes).

	sCXL (*n* = 29)				aCFXL (*n* = 25)			
	Mean ± SD		*p*-Value vs. Baseline		Mean ± SD		*p*-Value vs. Baseline	
Follow-up (months)	12 months	24 months	12 months	24 months	12 months	24 months	12 months	24 months
**Refractive parameters**								
Corrected distance acuity (logMAR)	−0.03 ± 0.013	−0.04 ± 0.015	0.04	0.02	−0.009 ± 0.004	−0.015 ± 0.005	0.05	0.012
Spherical equivalent (D)	0.38 ± 0.20	1.36 ± 0.53	0.12	0.02	0.6 ± 0.2	1.21 ± 0.37	0.012	0.006
Refractive cylinder magnitude (D)	0.36 ± 0.24	1.39 ± 0.53	0.28	0.02	0.95 ± 0.38	1.35 ± 0.46	0.02	0.014
**Topographical parameters**								
Maximum keratometry (D)	−0.78 ± 0.31	−0.97 ± 0.35	0.034	0.018	−0.99 ± 0.34	−1.1 ± 0.38	0.014	0.014
Mean K (D)	0.49 ± 0.2	0.49 ± 0.23	0.04	0.08	−0.58 ± 0.25	−0.59 ± 0.23	0.04	0.02
Minimum corneal thickness (μm)	−5.8 ± 4.1	−1.0 ± 3.5	0.2	1.0	−6.0 ± 5.5	−1.6 ± 2.9	0.4	1.0
**Biomechanical parameters**								
Corneal hysteresis (mmHg)	1.63 ± 0.5	1.09 ± 0.3	0.004	0.002	2.03 ± 0.78	1.94 ± 0.61	0.002	0.008

## Data Availability

The data presented in this study are available on request from the corresponding author. The data are not publicly available due to privacy and ethical reasons.
